# Ameliorative Effect of Linalool in Cisplatin-Induced Nephrotoxicity: The Role of HMGB1/TLR4/NF-κB and Nrf2/HO1 Pathways

**DOI:** 10.3390/biom10111488

**Published:** 2020-10-28

**Authors:** Maged E. Mohamed, Yamen S. Abduldaium, Nancy S. Younis

**Affiliations:** 1 Department of Pharmaceutical Sciences, College of Clinical Pharmacy, King Faisal University, Al-Ahsa 31982, Saudi Arabia; nyounis@kfu.edu.sa; 2 Department of Pharmacognosy, College of Pharmacy, Zagazig University, Zagazig 44519, Egypt; 3 Department of Orthopaedics, College of Medicine, Zagazig University, Zagazig 44519, Egypt; yamensafwat83@gmail.com; 4 Pharmacology Department, Zagazig University, Zagazig 44519, Egypt

**Keywords:** apoptosis, essential oil, human cell line cytotoxicity, monoterpenes, oxidative stress, toll-like receptors

## Abstract

Background: The monoterpene linalool is a well-known essential oil component produced by several aromatic plants. Cisplatin is a widely used anticancer drug that produces many side effects, particularly nephrotoxicity. Here, we aimed to inspect linalool’s protective activity against cisplatin-induced nephrotoxicity and explore part of the underlying mechanisms. Methods: Male Wistar rats were given linalool (50 and 100 mg/kg/day orally) for 15 days; then challenged with cisplatin (8 mg/kg) on the 12th day. Renal function parameters, oxidative stress, inflammatory and apoptotic markers, and toll-like receptor pathway gene, and protein expressions were investigated. Histopathology, immunohistochemistry, and cell-line mediated cytotoxicity assays were conducted. Results: Linalool ameliorated kidney function after cisplatin challenge and managed all oxidation system parameters including GSH, SOD, CAT, MDA, NADPH, and particularly the Nrf2-mediated pathway markers. Linalool decreased TLR4, MYD88 and TRIF gene and protein expressions; diminished related inflammatory mediators such as TNF-α, IL-1β, IL-6, and NF-κB; and down-regulated HMBG1. Linalool mitigated cisplatin-induced apoptotic markers such as caspase 3, caspase 9, and Bax expression, and boosted the anti-apoptotic Bcl2 expression. Linalool potentiated the cytotoxic effect of cisplatin when investigated on HeLa and PC3 human cancer cell lines. Conclusion: Linalool could protect against cisplatin-induced kidney function and tissue damage.

## 1. Introduction

**Figure 1:**
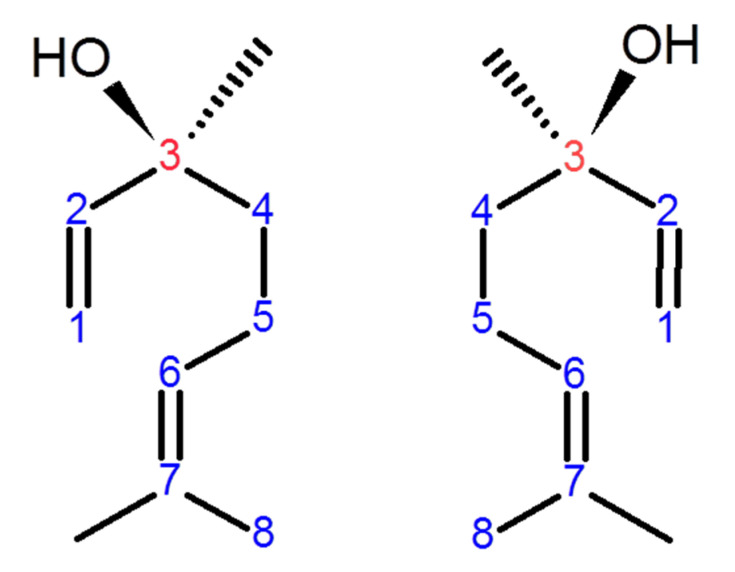
The stereo-configuration of linalool. (*S*)-(+)-linalool or coriandrol (**left**) and (*R*)-(–)-linalool or licareol (**right**). The enantiomeric properties of linalool are due to the chiral carbon number 3 (red-colored).

Linalool, a naturally occurring acyclic monoterpene alcohol, is found in numerous aromatic plant families such as Lamiaceae and Rutaceae. It is a constituent of numerous essential oils produced by many common and edible plants such as peppermint, coriander, and cinnamon [1]. Linalool naturally exists in two isomeric configurations depending on the chiral carbon number 3 (Figure 1). *S*-(+)-linalool, also called coriandrol, is the first enantiomer of linalool and it is characterized by an herbaceous smell. The second enantiomer is (*R*)-(−)-linalool, also called licareol, which possesses a lavender-like odor [2,3]. Linalool exists in the plant essential oil as pure S or pure R isoform or as a racemic mixture (SR); however, many studies demonstrated that the R isomers are more common than the S isomers [3,4]. Linalool possesses numerous pharmacological activities including neuroprotective [5], cardio-protective [6], hepatoprotective [7], antiapoptotic [1], antioxidant [5], anti-inflammatory [8,9,10], antihyperalgesia [8], antinociceptive [8], and anti-ulcerative colitis [11] activities. Due to its anti-inflammatory and antioxidant activities, linalool displays neuroprotection in hemi-Parkinson’s rats [5]. Linalool is a reducing agent due to its ability to scavenge ROS and to stimulate other antioxidants from their inactive state [1].

Linalool retains anticancer effects. It exerts significant actions in human oral cancer cells by prompting cell apoptosis and diminishing the phosphoinositide 3-kinases (PI3K)/protein kinase B (AKT) signaling pathway [12]. Linalool is a promising anticancer agent for hepatocellular carcinoma (HCC) therapy via inducing cell apoptosis via Ras, Mitogen-activated protein kinase (MAPKs), and the protein kinase B (AKT)/mammalian target of rapamycin (mTOR)pathways [13].

Linalool demonstrates anti-inflammatory action in various in vivo and in vitro studies, such as in UVB-activated Human Dermal Fibroblasts adult (HDFa) skin cells [1], lipopolysaccharide (LPS)-stimulated H292 airway epithelial cells [9], and ovalbumin-induced airway hyperresponsiveness [9]. Briefly, pre-treatment with linalool significantly inhibited UVB-induced phosphorylation of ERK1, JNK, and p38, with subsequent deterrence of nuclear factor-κB NF-κB translocation and the overexpression of TNF-α, IL-6, IL-10, COX-2, MMP-2, MMP-9, Bax, and p53 in HDFa skin cells [1]. Linalool inhibited inflammatory cells influx and mucus secretion in the lung tissues caused by ovalbumin exposure [9]. In addition; it downregulated nitric oxide synthase (iNOS) expression, AKT, MAPKs, and (NF-κB) signaling in the lung tissues [9]. 

Deepa and Venkatraman Anuradha [14] proved that linalool is a potential renal protective compound in diabetic patients via re-establishing glucose-metabolizing enzymes and GLUT-1 expression, and inhibiting nephron loss and rescuing kidney from oxidative stress and inflammation [14]. 

Cisplatin, a water-soluble and low-molecular-weight platinum-based drug, is widely used in the management of widespread types of cancers [15]. Nevertheless, its clinical worth and efficacy are restricted due to its side effects, such as ototoxicity, renal toxicity, and neurotoxicity, among which nephrotoxicity is the most alarming [15,16]. Cisplatin-induced nephrotoxicity essentially arises in proximal tubular epithelial cells and is characterized by tubular necrosis, oxidative destruction, inflammatory injury, and renal failure [16]. Currently, after cisplatin administration, 20–30% of patients display acute kidney injury (AKI) despite therapy improvements [15]. Cisplatin metabolizes into a highly reactive molecule that depletes renal epithelial cells’ natural antioxidant system, both the oxidative as well as the nitrosative systems, and interacts with numerous cellular constituents, causing both functional and structural protein damage, autophagy, apoptosis, and necrosis [15]. As a result of cisplatin administration, cytokines, various receptors, and leukocyte are either augmented or stimulated, leading to inflammation that contributes to renal damage [15].

Toll-like receptors (TLRs) are a family of receptors considered as the first line of innate defense by recognizing pathogen-associated molecular patterns as well as endogenous tissue injury signals [17]. Among TLRs, TLR4 appears to be a sensor for cisplatin-induced epithelial injury. Once activated, TLR4, on renal parenchymal cells, activates numerous pathways (such as p38 MAPK), leading to increased production of inflammatory cytokines, and subsequent kidney injury [18], therefore, in the current study, we are proposing TLR 4 pathway as one mechanism of action, through which linalool might exert this action.

Many natural products, phytochemicals, essential oils, food supplements, and nutraceuticals have been used to ameliorate the kidney damage induced by cisplatin. Many plant extracts, usually used in folk medicine preparation, improve cisplatin’s side effects, including nephrotoxicity, such as tea [19], ginseng [20], honey and royal jelly [21], grape seed [22], pomegranate rind [23], seed oil [24], and flower [25] extracts. Numerous single phytochemical components possess anti-cisplatin-induced nephrotoxic activity such as capsaicin [26], glycyrrhizic acid [27] and isoliquiritigenin [27], ginsenoside Rg5 [28], curcumin [29], β-caryophyllene [30], and carnosic acid [31]. Some essential oils exert a protective effect counteracting cisplatin-induced kidney toxicity: fennel [32] and *Pituranthos chloranthus* [33]. Many food supplements, vitamins, and other nutraceuticals mitigate cisplatin’s side effects, e.g., *Panax* saponins [34] and anthocyanins [35], grape seed proanthocyanidins [36], vitamin C [37], and riboflavin [38]. 

To the best of our knowledge, the effect of linalool has never been investigated against cisplatin-induced nephrotoxicity. Therefore, the main objective of this study was to examine the ameliorative efficacy of linalool on the cisplatin-induced nephrotoxicity status. The mechanism of action through which linalool might exert this action was scrutinized.

## 2. Materials and Methods 

### 2.1. Reagents and Kits 

(*R*)-(−)-linalool (synonym: licareol, *L*-linalool, Cat. No. W263516), cisplatin (synonym: *cis-*diammineplatium II dichloride, Cat. No. 15663-27-1) were obtained from Merck (Sigma Adrich, St. Louis, MO., USA). Blood urea nitrogen (BUN) and serum creatinine colorimetric assays were purchased from Biodiagnostic Co. (Cairo, Egypt). Reduced glutathione (GSH, Cat. No. 703202), malondialdehyde (MDA, Cat. No. 700870), catalase (CAT, Cat. No.707002), superoxide dismutase (SOD, Cat. No. 706002), and NADPH (Cat. No. 9000743) ELISA kits were attained from Cayman Chemicals (Ann Arbor, MI, USA). Cleaved caspase 3 (Cat. No. KHO1091), caspase 9 (Cat. No. BMS2025), Bcl-2 (Cat. No. ERBCL2L2), and Trizol reagent kit (Cat. No. 15596026) were acquired from Thermo Fisher Scientific (Carlsbad, CA, USA.). Bax (Cat. No.E4513) and heme oxygenase-1 (HO-1, Cat. No. E4525) were attained from BioVision (Zürich, Switzerland). RT-PCR kit (Cat. No. RR037A) and SYBR ExScript RT-PCR kit (Cat. No.RR014B) were purchased from TaKaRa, (Shiga, Japan). Tumor necrosis factor-α (TNF-α, Cat. No. ab46070), interleukin-1β (IL-1β, Cat. No. ab100768), interleukin-6 (IL-6, Cat. No. ab100772), and nuclear factor-κB (NF-κB, Cat. No. ab133112) ELISA kits were purchased from Abcam Co., Ltd. (Eugene, OR, USA). Toll-like receptors TLR4 (SC-10741), MyD88 (SC-11356), reference gene (β-actin; SC-130656), goat anti-rabbit immunoglobulin (Ig) and G-horseradish peroxidase (HRP) (SC-2030) antibodies, luminol reagent (SC-2048), and polyvinylidene fluoride (PVDF) membrane (SC-3723) were purchased from Santa Cruz Biotechnology, Inc. (Dallas, TX, USA). Additional chemicals and reagents used were of the uppermost analytical rank acquired from commercial sources.

### 2.2. Animals 

Rats (Wister male, weight 200–250 g) were supplied by the animal house facility, King Saud University, Riyadh (Kingdom of Saudi Arabia). The animals were kept under a 12 h. light/dark cycle with free access to food and water *ad libitum* at a temperature of 23 ± 2 °C for 1 week prior to the experiment. Food consisted of standard diet pellets contained not less than 20% protein, 5% fiber, 3.5% fat, 6.5% ash, and a vitamin mixture. Average measurements of body weight and food intake were conducted weekly during the study period. The bodyweight of the rat was measured before euthanasia. After anesthesia, the kidneys were surgically removed and weighed. All experiments were appropriately performed in accordance with the “Ethical Conduct for Use of Animals in Research” Guidelines in King Faisal University and the “Executive Regulations for Research Ethics on Living Creatures (Second Edition)”, published by National Bioethics Committee, Saudi Arabia. All animal care and experimental procedures were approved by the Animal Research Ethics Committee at King Faisal University (KFU-REC/2019-3-20). 

### 2.3. Experimental Design

Rats were allocated into five groups (n = 8) after the one-week acclimatization period in the facility: Group I was given 0.05% carboxymethyl cellulose in saline (0.9% NaCl) by oral gavage tubes for 14 days (Normal). Group II rats were administered linalool (100 mg/kg/day) suspended in 0.05% carboxymethyl cellulose in saline (0.9% NaCl) [5,14] orally for 14 days (Linalool). Group III was given 0.05% carboxymethyl cellulose in saline (0.9% NaCl) by oral gavage tubes for 14 days and on 12th day, the animals were given single dose of cisplatin (8 mg/kg, i.p.), which was freshly prepared in saline (0.9% NaCl) [39] (Cisplatin). Group VI and V rats were pre-treated with 50 and 100 mg/kg/day orally (as previous groups), respectively, for 14 days and were given cisplatin (8 mg/kg i.p.) on the 12th day (Cisplatin + linalool (50 or 100 mg/kg).

### 2.4. Blood and Tissue Samples Collection

Three days after cisplatin injection (day 15), the animals were euthanized using pentobarbitone sodium (60 mg/kg; i.p.) for blood and tissue collection. Blood was withdrawn from the heart, and it was allowed to clot for 2 h at room temperature then centrifuged (2500× *g*, 20 min) to isolate the serum, which was stored at –20 °C. Renal tissue samples were dissected, and then one kidney from each animal was homogenated in 10% phosphate buffer and stored at –20 °C for further analysis of markers and indicators. The other kidney was used in the histological and immunohistochemistry studies. 

### 2.5. Assessment of Renal Function

Renal function indicators, including BUN and serum creatinine, were colorimetrically determined in serum using specified kits according to the manufacturer’s protocols using spectrophotometer (LEICA UNISTAT^®^; Leica Inc., Allendale, New Jersey). Bodyweight (g) was measured, and relative kidney weight (kidney index) was calculated according to:

Kidney index = (kidney weight/total body weight) × 100(1)

### 2.6. Assessment of Oxidative Stress Markers

Reduced glutathione (GSH), superoxide dismutase (SOD), catalase (CAT), and NADPH-1 levels were evaluated in renal tissue using relevant ELISA kits according to the manufacturer’s instructions. The malondialdehyde (MDA) assay kit was used to assess lipid peroxidation via estimating thiobarbituric acid reactive substances (TBARS) level, which is expressed as malondialdehyde equivalents. 

### 2.7. Assessment of Nuclear Factor E2-Related Factor 2 (Nrf2) and Heme Oxygenase-1 (HO-1)

Renal tissue levels of both Nrf2 and HO-1 in different experimental groups were assessed via ELISA kits following the manufacturers’ instructions. 

### 2.8. Assessment of Toll-Like Receptor Pathway Gene Expression 

Levels of gene expression for the TLR pathway (*TLR 4*, *MYD88*, *TRIF*, *Bax*, *Bcl-2*, and *HMGB-1*) were determined via real-time PCR using the primers’ sequences mentioned in Table 1 according to a method described elsewhere [40]. In brief, RNA from renal tissue samples was extracted and purified using the Trizol reagent kit, and then its cDNA was reverse transcribed using reverse transcription-polymerase chain reaction (RT-PCR) kit according to the manufacturer’s protocol. Real-time PCR was performed using SYBR ExScript RT-PCR kit according to the manufacturer’s instructions. Quantification investigations were completed via Opticon-2 Real-time PCR reactor (MJ Research, Capital Court, Reno, NV, USA). Step PE Applied Biosystems (Waltham, MA, USA) software was used to investigate real-time PCR results. Target gene expressions were measured and interrelated to the reference gene (*β-actin*).

### 2.9. Assessment of the Toll-Like Receptor Pathway Protein Expressions 

Western blot was performed according to the method described previously [40] to assess TLR pathway protein expression for TLR4, MYD88, and TRIF. Briefly, renal tissue samples were mixed with RIPA buffer containing protease inhibitor; the extracted protein was measured using a Nano Drop Lite spectrophotometer (Thermo Fisher Scientific, Waltham, MA, USA). Thereafter, 50 µg of the total extracted protein was separated via SDS-PAGE and blotted onto PVDF membranes. PVDF membranes were blocked by incubation in TBS enclosing 3% bovine serum albumin and 0.1% Tween 20 for one hour at room temperature. PVDF membranes were washed (TBS containing 0.1% Tween 20), and incubated first with a 1:1000 dilution of the primary antibodies (TLR 4, MYD88, and TRIF) for two hours, and then with a 1:5000 dilution of the secondary antibody at room temperature. TLR4 (SC-10741), MyD88 (SC-11356), TRIF (SC-514384), reference gene (*β-actin*; SC-130656), goat anti-rabbit immunoglobulin (Ig) G-horseradish peroxidase (HRP) (SC-2030) antibodies were purchased from Santa Cruz Biotechnology, Inc. (Dallas, TEX, USA). The chemiluminescence produced was detected with the C-DiGit chemiluminescence scanner (LI-COR, Lincoln, NE, USA), and the band intensity was analyzed using the scanner software.

### 2.10. Assessment of Inflammatory Mediators

The effect of pre-treatment with linalool on cisplatin-induced kidney damage on the renal expression of the inflammation markers was investigated. Inflammation markers including TNF-α, IL-1β, IL-6, and NF-κB/p65 were measured via ELISA kits following the manufacturers’ instructions using a microplate reader SpectraMax i3X (Molecular devices). 

### 2.11. Assessment of Apoptotic Markers

The effect of cisplatin and linalool on kidney expression of the apoptotic markers, such as cleaved caspase 3 and caspase 9, was assessed using the specified ELISA kits following the manufacturers’ instructions using a microplate reader SpectraMax i3X (Molecular devices). 

### 2.12. Histopathological Investigation and Immunohistochemical Protein Assay

Kidney samples were fixed in 10% formalin for 24 h, and then paraffin beeswax tissue blocks were prepared for sectioning in 4 μm thicknesses by sledge microtome. The paraffin-embedded tissue sections were collected on glass slides, deparaffinized, stained by hematoxylin and eosin stain (H&E), periodic acid Schiff (PAS), and Masson (Masson’s trichrome) staining for examination under a light microscope for assessment of the histological injury. Renal injury was scored based on the proportion of damaged tubules in the kidney sample by a blinded pathologist observing ten consecutive non-overlapping fields per animal in a blinded manner.: 0 = normal kidney (no damage), 1 = minimal damage (less than 25% damage), 2 = mild damage (25–50% damage), 3 = moderate damage (50–75% damage), and 4 = severe damage (> 75% damage) as described previously [41]. 

For the immunohistochemical (IHC) staining, 3% hydrogen peroxide (H_2_O_2_) in methanol was used to block the endogenous peroxidase enzyme in the obtained sections at 21–25 °C for 30 min, followed by rinsing in PBS three times. Afterward, the sections were incubated with HMBG1, NF-κB, and TNF-α antibodies (1:100, Thermo Fisher Scientific, Cambridge, U.K.), which were then stored overnight at 4 °C in a humidified chamber, and then goat anti-rabbit-horseradish peroxidase (HRP) conjugated IgG antibody (1:1000; Cat. no. ab6721; Abcam, Eugene, OR, USA) was added for 1 h at 37 °C. Finally, the sections were developed with 1% diaminobenzidine for 5 min, counterstained with 1% hematoxylin for 2 min at 21–25 °C, and mounted with neutral gum. Kidney slices were imagined using a microscope fitted with a digital camera (Nikon Instruments Inc.). NIS-Elements software was used for quantitative analysis of HMBG1, NFκB and TNF-α. Firstly, the area of IHC reaction in the picture was selected. Secondly, the average optical density in the selected area of each picture was measured. Positive cells were counted under 400× magnification observing ten consecutive non-overlapping fields per animal in a blinded manner. 

### 2.13. Cell Lines Cytotoxicity Assay 

Cisplatin and linalool cytotoxic activities were determined via sulforhodamine B cytotoxicity assay as described elsewhere [42]. HeLa (cervical) and PC3 (prostate) human cancer cell lines were obtained from the American Type Culture Collection (ATCC) and maintained by serial sub-culturing. HeLa and PC3 cells were grown in RPMI-1640 medium, containing 10% (*v/v*) fetal bovine serum, 100 U/mL penicillin, and 100 μg/mL streptomycin antibiotics. After 24 hrs. of incubation at 37 °C in 5% CO_2_ and 95% air, the media were substituted with fresh ones containing different drug concentrations. Cisplatin was used at different concentrations ranging from 0.7 to 16 μg/mL, whereas linalool was used at concentrations of 100, 200, and 400 μM. Following treatment, cells were fixed with 10% trichloroacetic acid for 1 h at 4 °C. Cells were washed and stained with 50 μL 0.4% sulforhodamine B in 1% acetic acid for 30 min at 25 °C. Sulforhodamine B dye was solubilized with 100 μL 10 mM trizma^®^ base (pH 10.5). The optical density of each well was measured spectrophotometrically at 564 nm. Concentration–response curves were sketched and inhibitory concentration 50 (IC_50_) for each curve was determined using Graph Pad software (version 5, San Diego, CA, USA). 

### 2.14. Statistical Analysis 

Data are presented as mean ± SD. For multiple comparisons, one-way ANOVA followed by Tukey–Kramer as a post-hoc test was performed. The 0.05 level of probability was used as the significance level. All statistical analyses were performed using Graph Pad software (version 5, San Diego, CA, USA). 

## 3. Results

### 3.1. Renal Function

Table 2 shows the mortality rate, body weight change, kidney index, as well as renal function markers (BUN and creatinine). The cisplatin-administered group had the highest number of animal deaths, while no animal death was recorded in the control or in the linalool pre-treated groups. Cisplatin caused a bodyweight decrease together with a kidney index increase. BUN and creatinine are hallmarks of kidney function. Table 2 showed that compared with the control group, cisplatin at a dose of 8 mg/kg highly amplified the creatinine (from 0.38 ± 0.12 mg/dL to 3.21 ± 0.16mg/dL) and BUN (from 35.75 ± 3.7 mg/dL to 174.33 ±1 4.50 mg/dL) levels, demonstrating nephrotoxicity generation in the cisplatin-administered animals. Pre-treatment with linalool at doses of 50 and 100 mg/kg revealed a significant renal protection effect as demonstrated by the reduction in renal function BUN and creatinine compared with cisplatin administered animals.

### 3.2. Oxidative Stress Markers 

Table 3 demonstrates the GSH, SOD, CAT, MDA, and NADPH oxidase-1 in the different investigational groups. Observable depletion in the antioxidant ability was identified in the cisplatin-induced acute kidney injury group, as demonstrated by the significant reduction in GSH, SOD, and CAT levels compared to the control animals. MDA and NADPH oxidase-1 levels were significantly amplified in the cisplatin-injected group compared to the control group. The groups pre-treated with 50 and 100 mg/kg linalool showed significant increases in GSH, SOD, and CAT and declines in MDA and NADPH oxidase-1 compared to the cisplatin group. 

### 3.3. Nrf2/HO-1 Signaling Pathway

**Figure 2:**
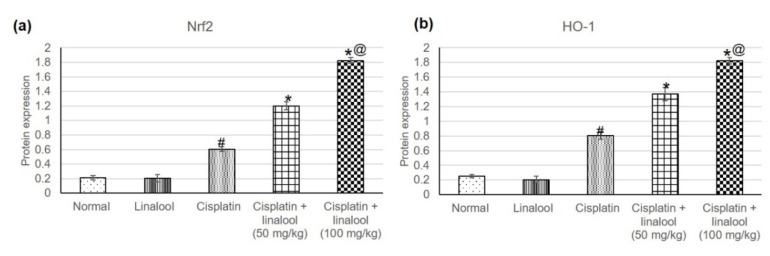
Effects of pre-treatment with linalool (50 and 100 mg/kg) for 14 days on the Nrf2/HO-1 signaling pathway in cisplatin-induced acute kidney injury: (**a**) Nrf2 and (**b**) HO-1 levels. All values are expressed as mean ± SD. #, statistically significant from normal group, *, statistically significant from cisplatin-only group, @, statistically significant from cisplatin + linalool 50 mg/kg group (*p* < 0.05) using one-way ANOVA followed by Tukey’s post-hoc analysis.

The assessment of Nrf2 and HO-1, Figure 2, revealed no significant differences in the two enzymes’ levels between the linalool and normal groups. Cisplatin significantly amplified the Nrf2 and HO-1 levels, reaching 0.60 ± 0.032 vs. 0.21 ± 0.03, and 0.80 ± 0.048 vs. 0.25 ± 0.020, respectively, when compared to the normal group. Pre-treatment with linalool at both doses prompted an extensive increase in Nrf2 and HO-1 compared to cisplatin-induced acute kidney injury. 

### 3.4. TLR Pathway Gene and Protein Expressions

**Figure 3:**
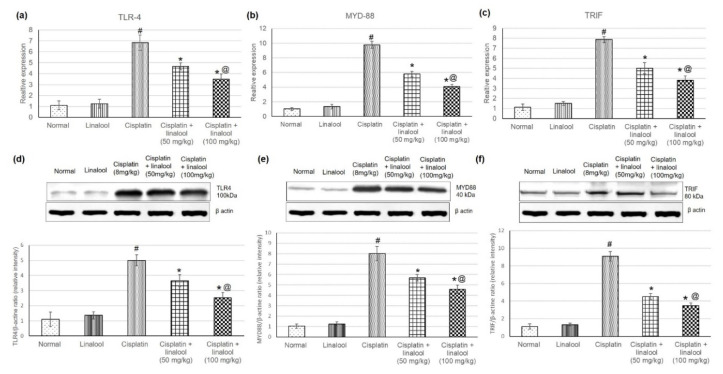
Effects of pre-treatment with linalool (50 and 100 mg/kg) for 14 days on the TLR pathway gene expression (mRNA amplification) (**a**) TLR4, (**b**) MyD88, and (**c**) TRIF, and TLR pathway proteins’ expression (**d**) TLR4, (**e**) MYD88, and (**f**) TRIF in cisplatin-induced acute kidney injury. All values are expressed as mean ± SD. # indicates statistically significant compared with the normal control group, * indicates statistically significant from the cisplatin-only group, and @ indicates statistically significant from the cisplatin + linalool 50 mg/kg group (*p* < 0.05) using one-way ANOVA followed by Tukey’s post-hoc analysis.

Figure 3 displays the quantitative PCR analysis of cisplatin-induced acute kidney injury and the effect of pre-treatment with linalool at 50 and 100mg/kg on the TLR pathway gene expressions. Real-time PCR results illustrated that cisplatin significantly increased TLR4 together with adaptor proteins MYD88 and TRIF expression. Whereas pre-treatment with linalool significantly (*p* < 0.05) diminished TLR4 expression compared with the cisplatin group, measuring 4.65 ± 0.35 and 3.18 ± 0.35 vs. 6.8 ± 0.37, respectively (Figure 3a). Similarly, pre-treatment with linalool (50 and 100 mg/kg) considerably lessened both MYD88 (reaching 5.79 ± 0.37 and 4.08 ± 0.28 vs. 9.77 ± 0.46, respectively) and TRIF (5.02 ± 0.56 and 3.93 ± 0.4 vs. 7.87 ± 0.63, respectively) relative expression levels compared to the cisplatin group (Figure 2b,c).

In addition, the effect of linalool pre-treatment on TLR pathway protein levels was evaluated through Western blot analysis of renal tissue proteins (Figure 3d–f) to determine whether linalool could suppress TLR protein production. Cisplatin prompted a substantial increase in the TLR proteins including TLR 4, MYD88, and TRIF compared with the normal control. Linalool alone had no influence on the TLR pathway protein content in the renal tissue compared to the normal group. Linalool pre-treatment at doses of 50 and 100 mg/kg diminished cisplatin-induced TLR protein escalation. 

### 3.5. Inflammatory Mediators

**Figure 4:**
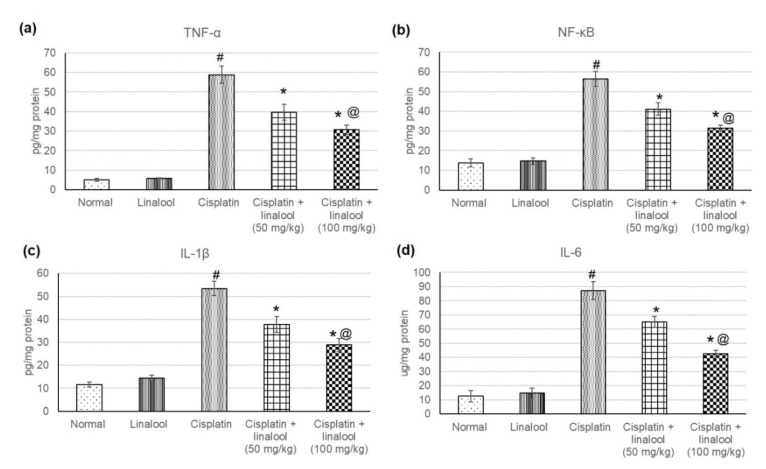
Effects of pre-treatment with linalool (50 and 100 mg/kg) for 14 days on the inflammatory mediators (**a**) TNF-α, (**b**) NF-κB, (**c**) IL-1β, and (**d**) IL-6 in cisplatin-induced acute kidney injury. All values are expressed as mean ± SD. # indicates statistically significant from normal control group, * indicates statistically significant from cisplatin-only group, and @ indicates statistically significant from cisplatin + linalool 50 mg/kg group (*p* < 0.05) using one-way ANOVA followed by Tukey’s post-hoc test.

The evaluation of different inflammatory mediators such as TNF-α, IL-1β, IL-6 and NF-κB verified the protective effects of linalool (50 and 100 mg/kg) against cisplatin-induced acute kidney injury (Figure 4). Cisplatin administration induced an augmentation in various inflammatory mediators, indicating severe inflammatory status, whereas no significant change was detected in animals administered linalool only. Linalool ingestion at a dose of 100 mg/kg prior to the cisplatin insult significantly decreased inflammatory mediators compared to linalool at a dose of 50 mg/kg, reaching 39.65 ± 4.01 vs. 30.68 ± 2.5 for TNF-α, 37.82 ± 3.4 vs. 28.84 ± 2.7 for IL-1β, 65.06 ± 3.8 vs. 42.56 ± 2.32 for IL-6, and 41.15 ± 3.12 vs. 31.34 ± 1.62 NF-κB. 

### 3.6. Apoptotic Markers 

**Figure 5:**
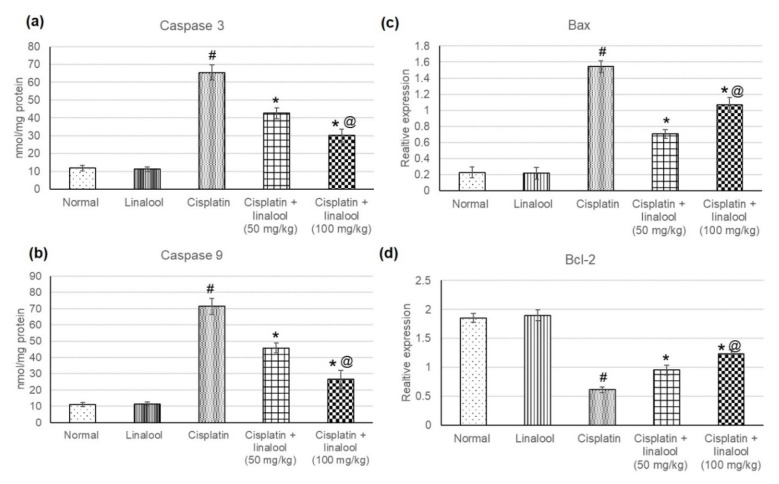
Effects of pre-treatment with linalool (50 and 100 mg/kg) on the apoptotic markers: (**a**) Caspase 3, (**b**) Caspase 9, (**c**) Bax, and (**d**) Bcl2 levels in cisplatin-induced acute kidney injury. All values are expressed as mean ± SD. # indicates statistically significant from the normal control group, * denotes statistically significant from the cisplatin-only group, and @ indicates statistically significant from cisplatin + linalool 50 mg/kg group (*p* < 0.05) using one-way ANOVA followed by Tukey’s post-hoc test.

The determination of different apoptotic enzymes’ levels, such as Caspase 3 and Caspase 9 proteins, and *Bax* and *Bcl-2* gene expression, evaluated the apoptotic effect of cisplatin on renal cells (Figure 5). No significant differences in Caspase 3, Caspase 9, Bax, and Bcl2 were evidenced with the administration of linalool only. After cisplatin injection, Caspase 3, Caspase 9, and Bax tissue levels were significantly higher; however, Bcl2 was substantially lower compared to the normal animals. Pretreatment with linalool resulted in a substantial reduction in Caspase 3, Caspase 9, and Bax together with a significant increase in Bcl-2. 

### 3.7. Histopathological Investigation

**Figure 6:**
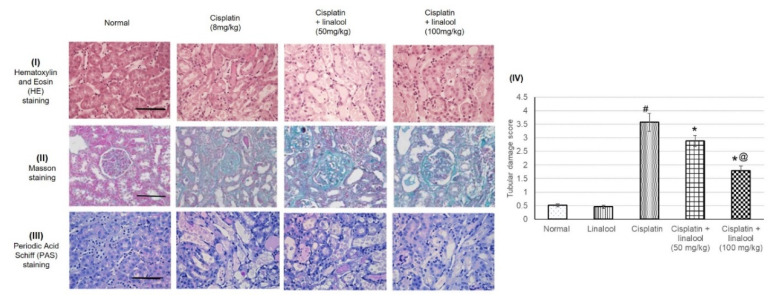
Histopathological analysis showing the protective effect of linalool (50 and 100 mg/kg) on the kidney tissues challenged through cisplatin injection: representative photomicrographs of (I) hematoxylin and eosin (H&E) staining, (II) Masson staining, and (III) periodic acid Schiff (PAS) staining, and (IV) quantitative assessment of tubular damage score. All values are expressed as mean ± SD. # indicates statistically significant from the normal control group, * denotes statistically significant from the cisplatin-only group, @ indicates statistically significant from the cisplatin + linalool 50 mg/kg group (*p* < 0.05) using one-way ANOVA followed by Tukey’s post-hoc test. Renal injury was scored based on the proportion of damaged tubules in the kidney sample where 0 = normal kidney (no damage), 1 = minimal damage (less than 25% damage), 2 = mild damage (25–50% damage), 3 = moderate damage (50–75% damage), and 4 = severe damage (> 75% damage).

H&E, Masson, and PAS staining showed that the rats experienced severe histological kidney injuries including a severe dilated tubular lumen, cell loss, brush border impairment, swelled renal tubular epithelial cell, degenerated necrotic proximal tubules, and tubular casts as a result of cisplatin administration (Figure 6). Pre-treatment with linalool diminished all these impairments, protected kidney tissue from damage, and significantly decreased the tubular damage score. 

### 3.8. Immunohistochemical Protein Assay

**Figure 7:**
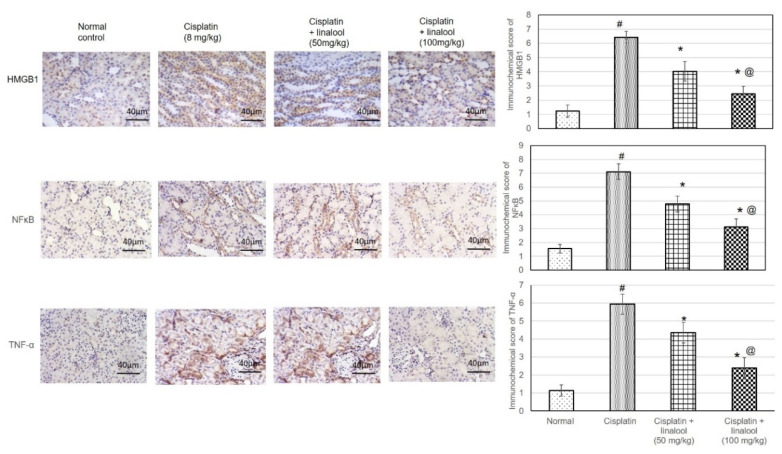
Immunohistochemical staining of renal tissues with HMBG1, NF-κB, and TNF-α antibodies after cisplatin induction of kidney injury. Pre-treatment with linalool (50 and 100 mg/kg) for 14 days diminished the number of positive cells containing HMBG1, NF-κB, and TNF-α. All values are expressed as mean ± SD (*n* = 3). # denotes statistically significant from the normal control group, * indicates statistically significant from the cisplatin-only group, and @ represents statistically significant from the cisplatin + linalool 50 mg/kg group (*p* < 0.05) using one-way ANOVA followed by Tukey’s post-hoc test.

As illustrated in Figure 7, immunohistochemical staining of the renal samples with HMBG1, NF-κB, and TNF-α antibodies produced only minimal immunoreactive responses in the normal and linalool groups. HMBG1-, NF-κB-, and TNF-α-positive cells were distributed widely in the cisplatin control renal sections. Sections from animals pre-treated with linalool had fewer positive cells. 

### 3.9. Cytotoxic Activity in HeLa and PC3 Human Cancer Cell Lines 

**Figure 8:**
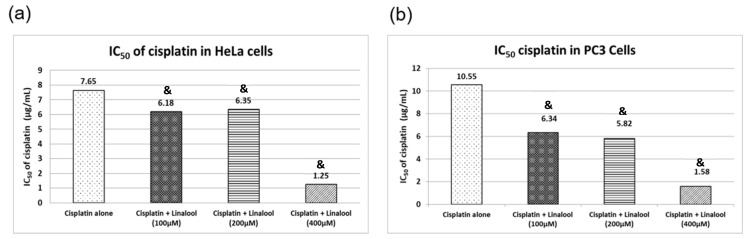
Cytotoxicity actions of c isplatin alone or in combination with different concentrations of linalool in HeLa (**a**) and PC3 (**b**) cancer cell lines. All values are expressed as mean ± SD. All values are expressed as mean ± SD. The symbol “&” indicates statistically significant from cisplatin-only group (*p* < 0.05) using one-way ANOVA followed by Tukey’s post-hoc test.

Figure 8 illustrates cell viability expressed as percentage survival of cancer cells using the sulforhodamine B cytotoxicity assay. Cisplatin induced substantial cytotoxic activity in HeLa and PC3 human cancer cells in a concentration-dependent manner, resulting in inhibitory concentration 50 (IC_50_) values of 8.90 and 10.55 μg/mL, respectively. Cancer cells treated with 100, 200, and 400 μM linalool for 24 hrs significantly boosted cisplatin cytotoxicity in a dose-dependent manner, while using 400 μM linalool reduced cisplatin IC_50_ significantly, reaching 1.25 and 1.58 μg/mL for HeLa and PC3 cancer cells, respectively (Figure 8a). The synergistic cytotoxic activity of linalool with cisplatin resulted in a concentration-dependent percentage decrease in the IC_50_ of Cisplatin by nearly 86% and 85% for HeLa and PC3 cancer cells, respectively (Figure 8b) when a dose of 400 μM linalool was used. Even when lower doses of linalool were used, i.e., 100 μM, the percentage decreases in the IC_50_ of cisplatin were 31% and 40% for HeLa and PC3 cancer cells, respectively (Figure 8b). 

## 4. Discussion

Nephrotoxicity is a substantial dose-preventive adverse effect induced by the clinical application of cisplatin. Therefore, new strategies for the prevention and management of cisplatin-induced kidney damage are fundamental. Although many natural products and phytochemicals have been used to mitigate cisplatin-induced nephrotoxicity, essential oils or their individual ingredients have not been scrutinized in detail. The main aim of this study was to examine the ability of linalool to protect renal function and tissues against cisplatin-induced damage.

### 4.1. Cisplatin and Oxidative Stress in Renal Tissues

In the current study, the cisplatin-administered group displayed bodyweight reduction, and kidney index, serum creatinine, and BUN levels escalation as well as severe histological injuries demonstrating nephrotoxicity. These results align with several previous studies that reported the toxic damaging effect of cisplatin on the kidney [15,16,42,43,44]. 

Several theories anticipated the underlying mechanisms responsible for this action of cisplatin on renal tissues [18,44,45]. One of the theories is the depletion of the antioxidant system [15,18], as cisplatin undergoes metabolic activation to highly reactive molecules [15]. Exhaustion or inactivation of antioxidants by cisplatin shifted the cellular redox status, causing endogenous ROS and oxidative stress accumulation inside the cells [44]. 

In this study, cisplatin obviously diminished the antioxidant ability of kidney tissues as shown from the reductions in the levels of GSH, SOD, and CAT, and elevations in MDA and NADPH oxidase-1 levels in the cisplatin group. 

### 4.2. Linalool Attenuation of Cisplatin-Induced Oxidative Stress in Renal Tissues

Linalool moderated oxidative stress in different experimental models. In the hereby study, pre-treatment with linalool produced a renal protective effect as demonstrated by the renal function improvement and histological injury reduction. Furthermore, linalool increased GSH, SOD, and CAT levels, and decreased MDA and NADPH oxidase-1, signifying that the compound attenuated oxidative stress via its powerful antioxidant activity. These effects of linalool are in accordance with several studies which illustrated linalool to be an antioxidant agent, for example, linalool showed protective activity against glutamate-induced oxidative stress in HT-22 neuronal cells and in an ex vivo model for glutamate excitotoxicity and NMDA toxicity [46]. Moreover, Linalool offered a neuroprotective effect in D-galactose- and aluminum trichloride (AlCl_3_)-induced Alzheimer’s disease (AD) via managing the oxidative stress in the hippocampus of AD mice [47]. Linalool inhalation improved antioxidative ability and reduced blood pressure in carpal tunnel syndrome (CTS) patients, which is characterized by peripheral neuropathy and ischemia–reperfusion damage [48].

### 4.3. The Nrf2 Mediated Oxidative Stress Pathway

Cisplatin affects the oxidation system through several pathways, one of which is the Nrf2 mediated oxidative stress response [45]. The transcription factor Nrf2 induces expression of cytoprotective genes, including NADPH: quinone oxidoreductase 1 (*Nqo1*) and heme oxygenase-1 (*Ho-1*) as a result of oxidative stress [47,49]. HO-1, and the products of heme degradation catalyzed by HO-1-sensitive antioxidant enzymes, prompt the expression of other antioxidant enzymes [47].

In this study, injection of cisplatin amplified Nrf2, and HO-1 levels, which is in agreement with previous studies that proved the detrimental role of Nrf2 and HO-1 [49]. For instance, Aleksunes, Goedken, Rockwell, Thomale, Manautou and Klaassen [49] proved that Nrf2-null mice exhibit widespread nephrotoxicity when challenged with cisplatin and a unique method to inhibit kidney damage is through Nrf2 pharmacological stimulation. Similar to HO-1, Pabla and Dong [44] demonstrated that HO-1-deficient mice are considerably more susceptible to cisplatin renal damage and that HO-1 overexpression reduces apoptosis induced by cisplatin. 

In the present study, linalool pre-treatment caused a further escalation in Nrf2, and HO-1 levels, instigating the Nrf2 pathway. Earlier studies found that linalool activated the Nrf2 signaling pathway, and thereby attenuated lung inflammation and oxidative stress induced by *Pasteurella multocida* [50]; mitigated cognitive deficits induced by Aβ [51]; amended acetic-acid-induced ulcerative colitis [11]; reduced LPS-induced inflammation in BV2 microglia cells [52]; and enhanced liver injury caused by LPS/d-galactosamine [53]. Thus, enhancing Nrf-2 and HO-1 antioxidant ability might play an imperative role in both the deterrence and handling of cisplatin-induced oxidative stress with subsequent kidney damage. 

### 4.4. TLRs and Their Tailoring and Ligand Markers

Another mechanism through which cisplatin produces such detrimental effects on the kidney is the TLR pathway. TLR4 receptor is expressed on the renal epithelium, and their expression is boosted with cisplatin challenge [54]. TLR pathway activation is responsible for initiating cytokine production and renal dysfunction during cisplatin nephrotoxicity, making renal parenchymal TLR receptors critical for mediating cisplatin renal toxicity [54]. 

In this study, cisplatin substantially amplified both the mRNA and protein expressions of TLR4, MYD88, and TRIF. Zhang, Ramesh, Uematsu, Akira and Reeves [54] revealed that cisplatin-treated wild-type mice demonstrated increased kidney impairment, histologic damage, and infiltrating leukocytes than equally-treated mice with a targeted deletion of TLR4 (*Tlr4*(−/−)]) [54]. 

Linalool diminished TLR4, MYD88, and TRIF mRNA and protein expressions. Therefore, linalool might be administered in diseases related to inflammation that is attributed to the TLR pathway overstimulation [10,55]. For instance, Lee, Wang, Li and Liu [10] found that endotoxin-induced expression of TLR4, MyD88, myeloid differentiation protein 2 (MD2), apoptosis-associated speck-like protein, and caspase-1 in spleen and mesenteric lymph nodes (MLNs) were all depressed by linalool administration [10]. 

To find the ligand for the TLR pathway in this study, HMBG1 was found within the renal sections obtained from the cisplatin control group as demonstrated from histoimmunochemistry analysis. This result aligns with previous reports that documented an increase in HMBG1 subsequent to cisplatin administration [16,56]. Cisplatin produces intracellular injury with subsequent release of damage-associated molecular pattern molecules (DAMPS) [57], which activate the TLR pathway during tissue injury [17]. Cisplatin contributes to HMGB1 release from kidney proximal tubular cells, which results in the promotion of inflammation during kidney damage [56]. However, sections from rats pre-treated with linalool displayed fewer HMBG1-positive cells, indicating downregulation. A previous study proved that linalool lowers endotoxin-induced HMGB-1 level in spleen and mesenteric lymph nodes (MLNs) [10]. 

### 4.5. Amelioration of Inflammatory Mediators 

TLRs release chemokines and other cytokines like TNF-α and IL-1β, attracting the inflammatory cells [57]. TLR4 is required for TNF-α production and subsequent nephrotoxicity after cisplatin injection, as the mediator (TLR4) is expressed in parenchymal cells rather than immune cells, suggesting that renal cells may be both the sensors of injury and producers of cytokines, which promote further injury [17].

In the current investigation, cisplatin administration induced an augmentation in several inflammatory mediators (TNF-α, IL-1β, IL-6, and NF-κB) indicting severe inflammatory status. This result is consistent with preceding studies that reported amplified TNF-α, IL-1β, IL-6, and NF-κB levels in the kidney tissues of cisplatin-treated rats [17,54,57]. Cisplatin is also accompanied by an escalation in IL-1β, IL-18, and IL-6, and neutrophil infiltration in the kidney [44]. 

Our results disclosed that linalool reduced inflammatory mediators TNF-α, IL-1β, IL-6, and NF-κB. This is in accordance with numerous reports describing the anti-inflammatory effects of linalool in streptozotocin-induced diabetic rats and on endotoxin-injected mice [10], glutamate- and NMD-induced neurotoxicity [46], ovalbumin-induced pulmonary inflammation [9], LPS-induced inflammation in BV2 microglia cells [52], in RAW 264.7 macrophages and LPS-induced lung injury [58], and carbon-tetrachloride-mediated hepatotoxicity [59]. Linalool lowered endotoxin-induced levels of IL-1β, IL-18, TNFα, IFN-γ, and HMGB1; the activation of NF-κB and caspase-1 in the spleen and mesenteric lymph nodes (MLNs) were also suppressed by linalool [10]. 

### 4.6. Linalool and Apoptosis

A third mechanism through which cisplatin may induce toxicity is renal cell apoptosis generation [18]. In the current study, caspase 3, caspase 9, and Bax tissue levels increased, while Bcl2 decreased substantially with cisplatin injection. 

Linalool pre-treatment attenuated cisplatin-induced caspase 3, caspase 9, and Bax expressions, and boosted the anti-apoptotic Bcl-2 expression, suggesting linalool protects the kidneys against cisplatin-induced apoptosis. Other studies found that management of linalool augmented Bcl-2 expression and depressed caspases 3 and 8 expressions in LPS/d-GalN-induced liver damage [53], and doxorubicin-induced cardiotoxicity [6]. Linalool pre-treatment prohibited UVB-mediated overexpression of proapoptotic indicators Bax and p53 in HDFa cells [1].

### 4.7. Linalool Effect on Cisplatin Potential as A Cytotoxic Agent 

The possible modulatory effect of linalool on cisplatin cytotoxic activity was inspected in vitro where two human cancer cell lines, HeLa and PC3, were used. Notably, we established that cisplatin induced significant cytotoxicity in both HeLa and PC3 human cancer cells. However, the presence of linalool boosted cisplatin cytotoxicity in a concentration-dependent manner by lowering the cisplatin inhibitory concentration (IC_50_) for HeLa and PC3 cancer cells. This boosting effect can be explained through previous reports that described the anti-cancer effect of linalool against human colon cancer [60], breast cancer cells [61], and epithelial ovarian carcinoma [62]. 

Linalool functions in a synergistic pattern with cisplatin and potentiates its cytotoxic activity and reduces its inhibitory concentration. Considering that linalool has rodent oral LD_50_ values of 2200 mg/kg in rats to 3500 mg/kg in mice [63], signifying its safety, suggests it might be used as a matrix for cisplatin injection; however, this approach needs further studies of the stability and interaction between linalool and cisplatin as well as the toxicity of linalool alone and with cisplatin in intravenous administration (the main route for cisplatin administration); further pharmacokinetic studies are also warranted. 

## 5. Conclusions

This study investigated the protective effect of linalool, the monoterpene alcohol, on renal function and tissues in a cisplatin-induced kidney injury rat model. Linalool succeeded, in part, in retaining normal kidney function and tissue integrity. Many mechanisms were suggested for linalool’s activity, including manipulation of the oxidation system, amelioration of TLR pathway genes and proteins’ expressions, and controlling cell apoptosis. Linalool improved all the measured oxidative parameters and more specifically, the Nrf2-mediated oxidative stress pathway markers (Nrf2 and HO-1) and nearly maintained their normal values after cisplatin challenge. Linalool successfully diminished TLR4, MYD88, and TRIF mRNA and protein expressions; decreased the production of related inflammatory mediators, including TNF-α, IL-1β, IL-6, and NF-κB; and down-regulated HMBG1 production. Linalool pre-treatment attenuated the expression of cisplatin-induced apoptotic markers such as caspase 3, caspase 9, and Bax, and boosted the anti-apoptotic Bcl-2 expression. Linalool did not reduce the cytotoxic effect of cisplatin when tried on human cancer cell lines; conversely, linalool, at a dose of 400 µM, potentiated this cytotoxic effect and significantly reduced the IC_50_, suggesting a reduction in cisplatin dose. Following all these mechanisms, linalool could protect the kidneys and its tissues against the side effects and potentiate the anticancer effect of cisplatin.

## Figures and Tables

**Table 1:** Primers sequence used for real-time PCR of toll-like receptor (TLR) pathway gene expression.

TLR Pathway Mediators	Primer Sequence (5′ to 3′)	
	Forward Primer	Reverse Primers
*TLR4*	AGTGTATCGGTGGTCAGTGTGCT	AAACTCCAGCCACACATTCC
*MyD88*	GAGATCCGCGAGTTTGAGAC	CTGTTTCTGCTGGTTGCGTA
TRIF	TCAGCCATTCTCCGTCCTCTTC	GGTCAGCAGAAGGATAAGGAA
*Bax*	AGACACCTGAGCTGACCTTGGA	CGCTCAGCTTCTTGGTGGAT
*Bcl2*	GGGATGCCTTTGTGGAACTATATG	CAGCCAGGAGAAATCAAACAGA
*HMGB-1*	AGGCTGACAAGGCTCGTTATG	TGTCATCCGCAGCAGTGTTG
*β-Actin*	CACGATGGAGGGGCCGGACTCATC	TAAAGACCTCTATGCCAACACAGT

**Table 2:** Effects of linalool and cisplatin on renal function markers in rats.

Treated Groups	Mortality Rate	Body Weight (gm)		Kidney Index	Blood Urea Nitrogen (BUN) (mg/dL)	Creatinine (mg/dL)
		Before Treatment	After Treatment			
Normal	0/10	190 ±8.68	220 ±10.75	0.71 ± 0.05	35.75 ± 3.7	0.38 ± 0.12
Linalool	0/10	200 ±11.46	240 ±12.47	0.74 ± 0.12	37.75 ± 2.82	0.46 ± 0.85
Cisplatin	2/10	189.42 ± 9.42	150.42 ± 7.46 ^#^	0.94 ± 0.05 ^#^	174.33 ± 14.50 ^#^	3.21 ± 0.16 ^#^
Cisplatin + linalool (50 mg/kg)	0/10	196.00 ± 14.75	185.00 ± 12.76	0.57 ± 0.10 *	70.83 ± 2.40 *	1. 53 ± 0.05 *
Cisplatin + linalool (100 mg/kg)	0/10	184.00 ± 13.5	178.00 ± 13.7	0.55 ± 0.08 *	67.83 ± 2.40 *	1.46 ± 0.05 *

All values are expressed as mean ± SD. #, statistically significant from normal control group; *, statistically significant from cisplatin-only group (*p* < 0.05 using one-way ANOVA followed by Tukey’s test as a post-hoc analysis.

**Table 3:** Effects of linalool and cisplatin on the markers of oxidative stress in kidney tissues of rats.

Treated Groups	GSH (μmol/g Tissue)	SOD (U/mg Protein)	CAT (nmol/g Tissue)	MDA (nmol/g Tissue)	NADPH (ng/mg Protein)
Normal	2.91 ± 0.12	16.22 ± 2.84	22.70 ± 0.66	12.20 ± 0.96	1.39 ± 0.35
Linalool	2.75 ± 0.26	19.48 ± 1.99	24.03 ± 0.87	14.43 ± 0.81	1.77 ± 0.67
Cisplatin	0.56 ± 0.08 ^#^	3.06 ± 0.69 ^#^	83.23 ± 5.34 ^#^	92.83 ± 6.17 ^#^	9.24 ± 0.88 ^#^
Cisplatin +linalool (50 mg/kg)	1.97 ± 0.31 *	11.03 ± 1.04 *	34.40 ± 6.6 *	34.96 ± 3.04 *	2.46 ± 0.72 *
Cisplatin +linalool (100 mg/kg)	2.27 ± 0.51 *^,@^	13.87 ± 1.31 *^,@^	28.65 ± 3.73 *^,@^	30.64 ± 2.15 *	2.01 ± 0.68 *

All values are expressed as mean ± SD. #, statistically significant from normal control group, *, statistically significant from cisplatin -only group, @, statistically significant from cisplatin + linalool 50 mg/kg group (*p* < 0.05) using one-way ANOVA followed by Tukey’s test as a post-hoc analysis.
